# LDH-A inhibitors as remedies to enhance the anticancer effects of PARP inhibitors in ovarian cancer cells

**DOI:** 10.18632/aging.203780

**Published:** 2021-12-16

**Authors:** Jiangdong Xiang, Lina Zhou, Yinyan He, Sufang Wu

**Affiliations:** 1 Department of Obstetrics and Gynecology, Shanghai General Hospital, Shanghai Jiaotong University School of Medicine, Shanghai 200080, P.R. China

**Keywords:** LDH-A, PARP inhibitors, BRCA1, ovarian cancer, proliferation

## Abstract

Ovarian cancer is one of the most lethal gynecologic malignancies. It has been shown that PARP inhibitors can selectively target BRCA-mutated ovarian cancer and exert some effects on ovarian cancer without BRCA mutations. However, the mechanism is still unclear. In this study, wild-type BRCA ovarian cancer cells (A2780 and SKOV3) were used. Our results showed that using a PARP inhibitor (olaparib or AG14361) alone significantly inhibited the proliferation of A2780 cells but negligibly inhibited the proliferation of SKOV3 cells. We used RNA sequencing to explore differentially expressed genes and found that PARP inhibitors increased LDH-A in SKOV3 cells, which was confirmed by RT-PCR. Oxamate (a specific inhibitor of LDH-A) was used to investigate whether LDH-A inhibition enhances the suppressive effects of PARP inhibitors on ovarian cancer without BRCA mutations. CCK-8 assays, scratch assays and Transwell assays were used to determine cell proliferation, cell migration ability and invasion ability, respectively. Both olaparib and AG14361 significantly inhibited the proliferation/invasion ability of A2780 cells but not SKOV3 cells. Inhibition of LDH-A can remarkably promote the inhibitory effects of PARP inhibitors on both A2780 and SKOV3 cells. Thus, high expression level of LDH-A influenced the suppressive effects of PARP inhibitors on ovarian cancer with wild-type BRCA, and LDH-A inhibition notably enhanced this effect.

## INTRODUCTION

Ovarian cancer is the fifth most lethal cancer type among women worldwide and is the leading cause of death from gynecologic malignancies [1]. A woman’s risk of developing ovarian cancer during her lifetime is approximately 1 in 78, and the risk of dying from ovarian cancer is approximately 1 in 108 [2]. It is believed that PARP inhibitors could be used to potentiate chemotherapy, and several PARP inhibitors are being evaluated for use in ovarian cancer. The multifunctional enzyme PARP plays an important role in DNA damage repair and genome stability, and in preclinical and clinical studies, PARP inhibitors have been found to restrain DNA repair pathways and induce the apoptosis of cancer cells with deficiencies in HR-mediated DNA repair, such as those carrying BRCA mutations [3]. The effect of PARP inhibitors on BRCA1 has been cited as a successful example of therapeutic ‘synthetic lethality' [4]. However, homozygous mutations of the BRCA genes significantly influence the cell response to PARP inhibitors [5]. Thus, there is an urgent need for the development of novel successful strategies to improve PARP inhibitor efficiency and ovarian cancer patient outcomes. The PRIMA trial revealed that patients with and without BRCA mutations can benefit from PARP inhibitors [6]. However, the risk of disease progression or death of the patients with BRCA mutations reduced by PARP inhibitors was to a significantly greater extent than that of patients without BRCA mutations [7].

BRCA1 plays a critical role in the regulation of homologous recombination (HR)-mediated DNA double-strand break repair. PARP activity is important for the chromatin changes required for efficient DNA repair [8]. When both pathways are simultaneously dysregulated, cells are unable to maintain sufficient DNA integrity and undergo mitotic catastrophe [9]. Cancer cells harboring BRCA1/2 mutations are sensitive to PARP inhibitors [10]. Approximately one-half of ovarian cancers harbor homologous recombination deficiencies (HRDs), and BRCA1/2 mutations account for half of these deficiencies [11]. To date, three PARP inhibitor drugs have been approved by the FDA in the United States for treating ovarian cancer treatment, namely, olaparib, rucaparib, and niraparib. The PRIMA trial, the first phase III prospective randomized clinical trial of PARP inhibitor monotherapy for first-line maintenance treatment in the whole population, which confirmed the benefits of niraparib, showed that patients with or without BRCA mutations acquired progression-free survival (PFS) benefits [6, 12]. The risk of disease progression or death of patients without BRCA mutations was reduced by 42%, a value significantly lower than that of patients with BRCA mutations (73%). Therefore, enhancing the efficacy of PARP inhibitors in the population without BRCA mutations will provide great benefit by improving the prognoses of ovarian cancer patients [13, 14].

Lactate dehydrogenase-A (LDH-A) has been shown to act as the key enzyme in the glycolytic pathway by catalyzing the interconversion of pyruvate and lactate and plays a critical role in tumor maintenance. Enhanced expression of LDH-A has been found to be associated with the evolution of aggressive and metastatic tumor types [15]. In line with these observations, our previous study suggested that the expression of LDH is significantly increased in ovarian cancer [16]. The main function of LDH-A is the conversion of pyruvate to lactate, which plays a key role not only in the energy metabolism pathway but also in DNA repair [17, 18]. LDH-A is a vital metabolic enzyme that is associated with cancer development, invasion, and metastasis. Researchers have shown that inhibition of LDH-A can enhance the sensitivity of drug-resistant cancers to other chemical drug treatments [19]. In our previous study, we observed that serum LDH was upregulated in ovarian cancer and was associated with aggressive tumor behavior. High LDH expression was statistically and positively correlated with the stage, pathological grade, and lymphatic metastasis of ovarian cancer patients [20]. Therefore, we hypothesize that LDH, especially LDH-A may affect the suppressive effect of PARP inhibitors on ovarian cancer cell lines without BRCA mutations.

In this study, we found that some kinds of ovarian cancer cells were insensitive to PARP inhibitors and further explored the specific mechanisms. We show, for the first time, that inhibition of LDH-A can notably enhance the inhibitory effects of PARP inhibitors on ovarian cancer with wild-type BRCA, which could be considered as a novel treatment.

## RESULTS

### The ovarian cancer cell line, SKOV3, was not sensitive to PARP inhibitors

**Figure 1:**
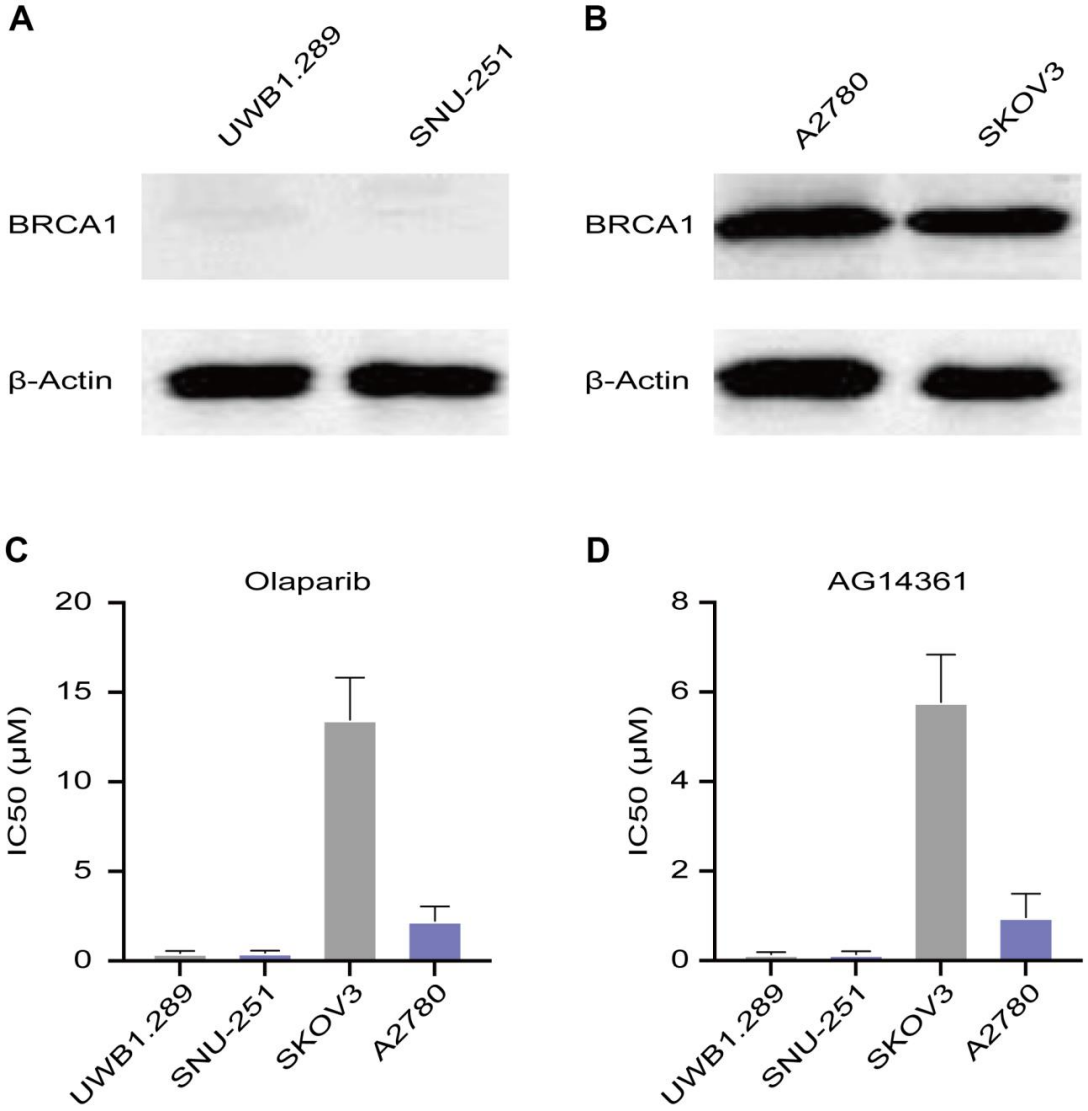
**Sensitivity of ovarian cancer cell lines to PARP inhibitors.** (**A**) Western blotting images of BRCA1 protein levels in UWB1.289 and SNU-251 cells. (**B**) BRCA1 protein levels in A2780 and SKOV3 cells were tested by western blotting assays. (**C**, **D**) Sensitivity of olaparib and AG14361 to ovarian cancer cell lines were represented by the IC50 values.

A2780 and SKOV3 cells are wild-type BRCA cancer cells, while UWB1.289 and SNU-251 cells have deleterious BRCA1 mutations. To verify these characteristics, we tested BRCA1 protein levels in these ovarian cancer cell lines. Western blotting assays confirmed that UWB1.289 and SNU-251 cells were BRCA1-mutated cells (Figure 1A). A2780 and SKOV3 cells expressed similar equal amounts of BRCA1 protein (Figure 1B). To test the sensitivity of ovarian cancer cells to PARP inhibitors, we treated these cells with different doses of PARP inhibitors (olaparib and AG14361). UWB1.289 and SNU-251 cells showed greater sensitivity to PARP inhibitors than compared to A2780 and SKOV3 cells, whereas SKOV3 cells showed the lowest sensitivity to both olaparib and AG14361 (Figure 1C, 1D). These results indicated that PARP inhibitor sensitivity is related not only to BRCA1 mutation but also to other latent factors.

### PARP inhibitor treatment increased LDH-A levels in SKOV3 cells

**Figure 2:**
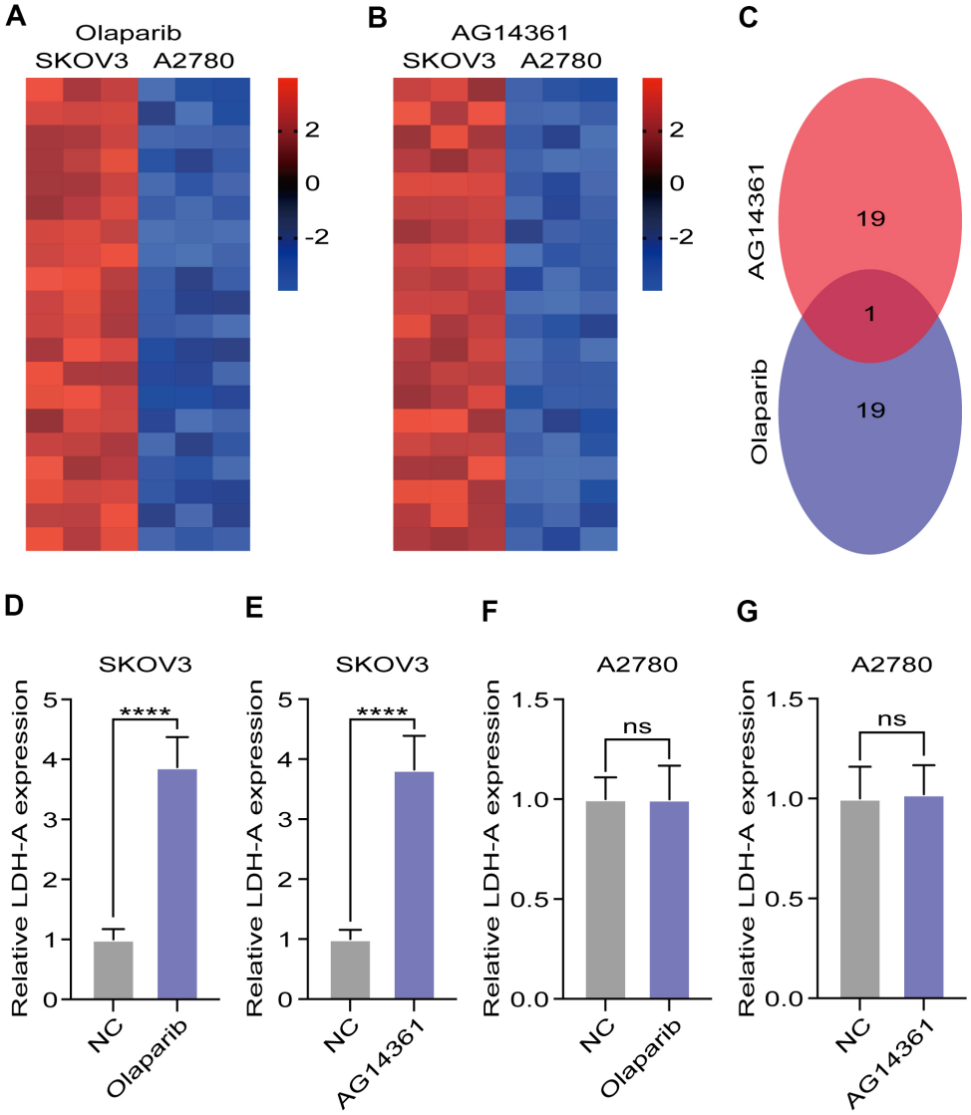
**LDH-A were increased in SKOV3 with PARP inhibitors treatment.** (**A**, **B**) Heat map demonstrated the differential genes in the RNA sequencing after PARP inhibitors treated for 24 hours. (**C**) Venn digram showed the intersection of differential genes in olaparib and AG14361 treated cells. (**D**–**G**) LDH-A levels in A2780 and SKOV3 were tested by RT-PCR after olaparib or AG14361 treated for 24 hours. Mean ± SEM, ****P < 0.001, ns: no significance.

To explore the potential reason for insensitivity to PARP inhibitors, we compared differentially expressed genes in A2780 and SKOV3 cells after olaparib or AG14361 treatment for 24 hours. The heatmap presents the dysregulated genes in the RNA sequencing data (Figure 2A, 2B). The Venn diagram shows that there was only one gene, LDH-A, existed at the intersection of the olaparib-treated group and the AG14361-treated group (Figure 2C). To verify these results, we tested LDH-A levels in A2780 and SKOV3 cells by RT-PCR after olaparib or AG14361 treatment for 24 hours. As shown, PARP inhibitor treatment increased LDH-A levels in SKOV3 cells (Figure 2D, 2E), while LDH-A levels did not significantly change with PARP inhibitors treatment in A2780 (Figure 2F, 2G). These results indicated that PARP inhibitors increased LDH-A levels in SKOV3 cells but not in A2780 cells, suggesting that LDH-A is a potential factor in reducing PARP inhibitor sensitivity.

### Oxamate suppressed the proliferation, migration, and invasion of both A2780 and SKOV3 cells

**Figure 3:**
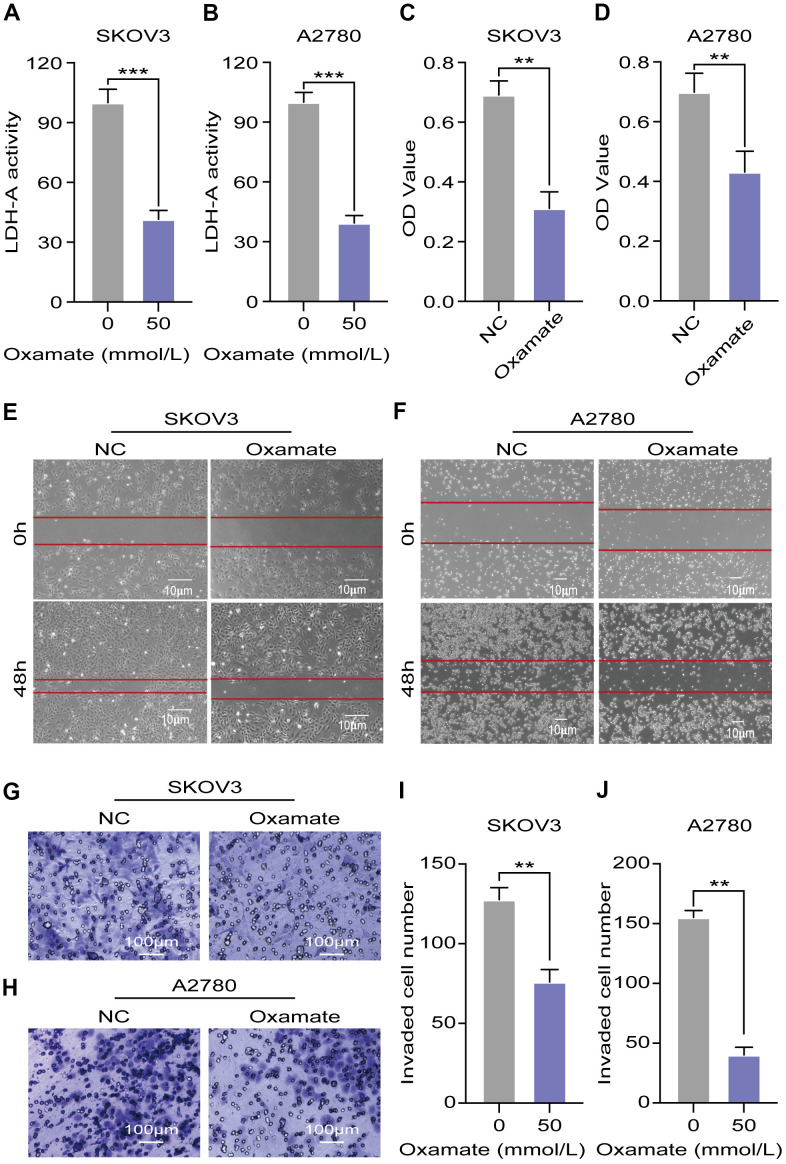
**Oxamate suppressed the proliferation, migration, and invasion ability of both A2780 and SKOV3 cells.** (**A**, **B**) Expression of LDH-A in A2780 and SKOV3 cells after oxamate treated for 24 hours. (**C**, **D**) Proliferation rates of A2780 and SKOV3 cells were evaluated by CCK-8 assays. (**E**, **F**) Scratch assays were used to test migration ability of A2780 and SKOV3 cells after oxamate treated for 48 hours. (**G**, **H**) Images of metastatic tumor cells were recorded by microscope. (**I**, **J**) Number of invaded cells were counted under the microscope. Mean ± SEM, **P < 0.01, *** P < 0.005.

To confirm the efficiency of the specific LDH-A inhibitor, we tested the LDH-A levels in A2780 and SKOV3 cells after oxamate treatment for 24 hours. The results showed that LDH-A levels were significantly decreased in both A2780 and SKOV3 cells (Figure 3A, 3B). To verify the effect of oxamate on ovarian cancer progression, we tested the proliferation, migration, and invasion ability of A2780 and SKOV3 cells. CCK-8 assays showed that oxamate reduced the proliferation rates of both A2780 and SKOV3 cells (Figure 3C, 3D). Regarding migration ability, scratch assays indicated that oxamate markedly reduced the migration ability of A2780 and SKOV3 cells (Figure 3E, 3F). We also tested the invasion ability of the SKOV3 and A2780 cells by Transwell assays. We captured metastatic tumor cells and counted them under a microscope. The results showed that oxamate effectively blocked the invasion ability of A2780 and SKOV3 cells (Figure 3G–3J). These results indicated that the proliferation, migration, and invasion of both A2780 and SKOV3 cells can be inhibited by the specific LDH-A inhibitor oxamate.

### Oxamate enhanced the tumor suppression effect of PARP inhibitors on ovarian cancer cells

**Figure 4:**
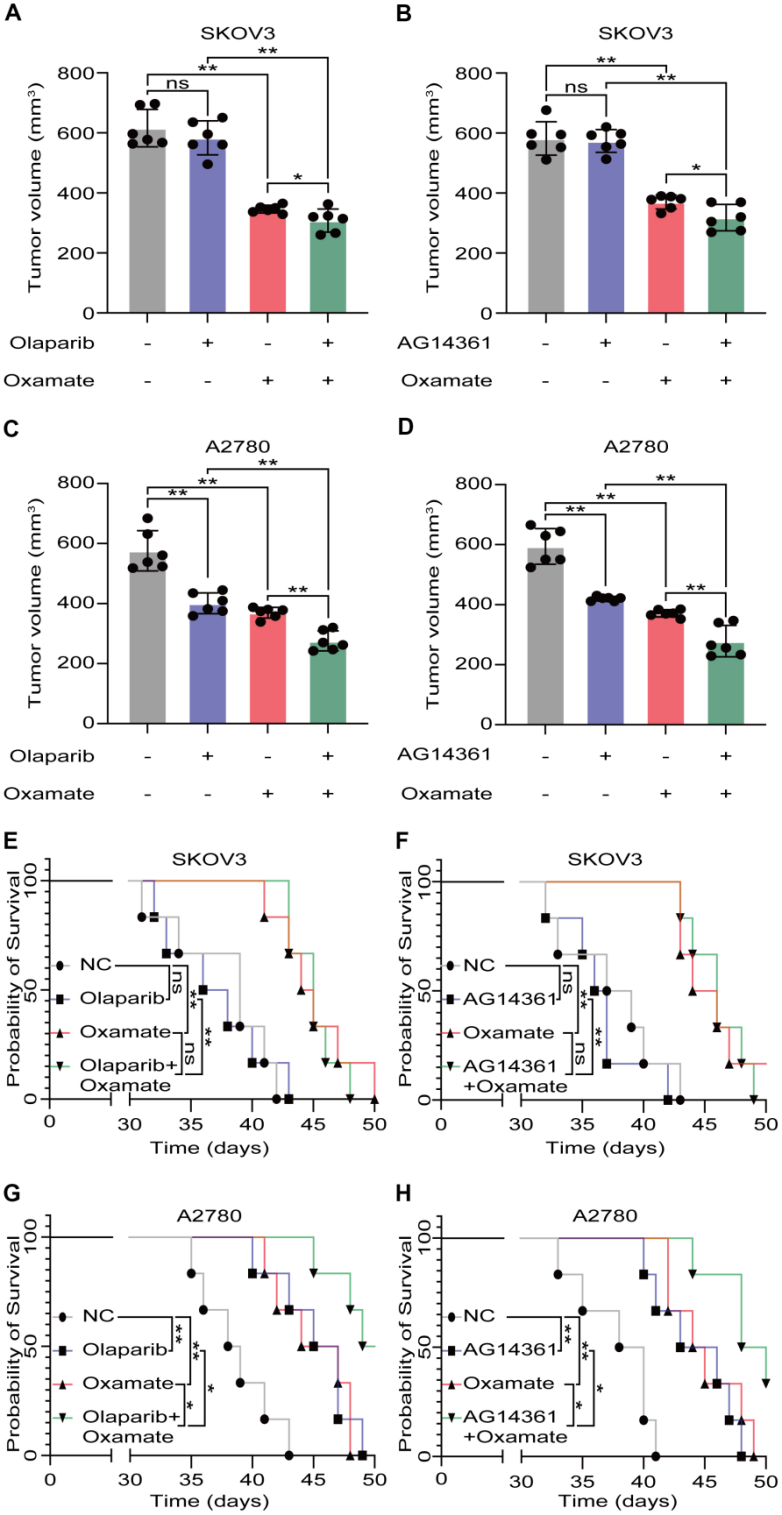
**Oxamate enhanced the tumor suppression effect of PARP inhibitors on ovarian cancer cells.** (**A**–**D**) Tumor volumes of xenograft tumor with oxamate and/or PARP inhibitors (olaparib or AG14361) treatment. (**E**–**H**) Survival time of xenograft tumor models with oxamate and/or PARP inhibitors (olaparib or AG14361) treatment were recorded. Mean ± SEM, *P < 0.05, **P < 0.01, ns: no significance.

Based on our previous findings, we next aimed to investigate whether a combination treatment including PARP inhibition and LDH-A inhibition showed synergistic effects *in vivo*. We treated SKOV3 and A2780 cells with oxamate and/or a PARP inhibitor (olaparib or AG14361) respectively and recorded tumor progression. Analysis of the volumes of xenograft tumors showed that the PARP inhibitors had obvious effects on A2780-derived xenograft tumors, but the effects on SKOV3-derived xenograft tumors were not significant. For the combination of oxamate and PARP inhibitor treatment, there was a synergistic anticancer effect on both SKOV3-derived xenograft tumors and A2780-derived xenograft tumors (Figure 4A–4D). The prognoses of xenograft tumor models were recorded, and the results showed that although the PARP inhibitors (olaparib and AG14361) did not significantly prolong the survival times of the animals with SKOV3-derived xenograft tumors, oxamate compensated for this shortcoming (Figure 4E, 4F). For the animals with A2780-derived xenograft tumors, combination treatment achieved the best effect (Figure 4G, 4H). These results indicated that even when the effects of PARP inhibitors are not ideal, the combined application of oxamate can effectively inhibit tumor progression and improve tumor prognosis.

## DISCUSSION

The emergence of PARP inhibitors brought new hope, but only 10-20% of patients with ovarian cancer carry BRCA1/2 mutations, and the need for a first-line maintenance treatment has not been met [21]. Therefore, methods to enhance the therapeutic efficacy of PARP inhibitors for patients without BRCA mutations are very urgently needed. In the current study, we first found that the two wild-type BRCA cell lines (SKOV3 and A2780) have very different responses to PARP inhibitors. Researchers have confirmed that the two cell lines do not have BRCA mutations and pointed out but they are the most frequently used and together account for 60% of publications on human ovarian serous cystadenocarcinoma cell line panels [22]. Our results showed that using olaparib or AG14361 alone significantly inhibited the proliferation of A2780 cells but negligibly inhibited the proliferation of SKOV3 cells. Neither cell line carries a BRCA mutation, but the therapeutic effects of PARP inhibitors were extremely different. To explore the potential reason, we compared differential genes in A2780 and SKOV3 after olaparib or AG14361 treated, and find only LDH-A existed in the intersection of the two groups. The results demonstrated that PARP inhibitors significantly increased LDH-A level in SKOV3 but not in A2780. It prompted that LDH-A was a potential factor in reducing PARP inhibitors sensitivity.

Numerous studies have demonstrated that LDH-A has aberrantly high expression in multiple cancers and is associated with malignant progression [23, 24]. Researchers have also revealed that LDH-A activation confers preinvasion, anti-anoikis and premetastatic advantages to cancer cells [25]. Consistent with these findings, our previous study revealed that LDH was significantly upregulated in ovarian cancer and was positively associated with the stage, pathological grade, and lymphatic metastasis [16, 20]. Therefore, LDH inhibitors are actively searched to be tested as potential anticancer agents [26]. Despite the discovery of LDH inhibitors with drug-like properties seems a hardly resolvable challenge, LDH inhibitors still show broad application prospects in clinic. In a recent published paper, lactate was found to affect the membrane potential of neurons and oxamate was found to suppress seizures in two animal models of epilepsy [27]. Researchers also found LDH inhibitors could alleviate the symptoms of the disease in animal models of autoimmune and allergic conditions [28]. Another recent study showed that LDH inhibition by oxamate caused a remarkable reduction of virus yield relevant for human pathology, without significant toxicity in host cells [29]. These data encouraged pharmaceutical industries and academic institutions in the search of small-molecule inhibitors of LDH.

In line with previous studies, the current research showed that using oxamate to inhibit LDH-A can significantly inhibited the proliferation, migration, and invasion of the two ovarian cancer cell lines expressing wild-type BRCA [30]. Some researchers have shown that inhibition of LDH-A can enhance the sensitivity of drug-resistant cancers to other chemical drug treatments [31, 32]. We further found that when LDH-A was inhibited, the proliferation of both SKOV3 and A2780 cells was markedly restrained by olaparib. The suppressive effect of PARP inhibitors on ovarian cancer cells without BRCA mutations were indeed enhanced by LDH-A inhibition. Combined with the results *in vivo*, these results indicated that additional mechanisms other than LDH-A-related mechanisms are associated with the effects of PARP inhibitor treatment. Our study suggested that for some patients, PARP inhibitors had poor efficacy in improving the prognosis even though tumors were slowly restricted.

Our results revealed that the two ovarian cancer cell lines harboring wild-type BRCA had very different responses to PARP inhibitors. The most important discovery of the current research was that the abrogation of LDH-A resulted in enhanced sensitivity to PARP inhibitors. To the best of our knowledge, this is the first discussion on enhancing the suppressive effects of PARP inhibitors on ovarian cancer without BRCA mutation.

## CONCLUSIONS

In this study, we found that the high expression level of LDH-A can significantly attenuate the inhibitory effects of PARP inhibitors on ovarian cancer without BRCA mutations. Combined treatment with LDH-A and PARP inhibitors represents a promising therapeutic approach for the treatment of ovarian cancer. Our valuable results may provide a new therapeutic strategy to treat patients without BRCA mutations.

## MATERIALS AND METHODS

### Cell lines and drugs

The human ovarian serous cystadenocarcinoma cell lines UWB1.289, SNU-251, A2780 and SKOV3 were purchased from the American Type Culture Collection (ATCC; Manassas, VA, USA) and maintained in RPMI-1640 medium supplemented with 10% fetal bovine serum (FBS), 100 U/ml penicillin, and 100 μg/ml streptomycin (all from Invitrogen; Thermo Fisher Scientific, Inc., Waltham, MA, USA) in a humidified incubator with 5% CO_2_ at 37° C. Oxamate (a specific LDH-A inhibitor), AG14361 (a specific PARP1 inhibitor) and olaparib (a PARP1/2 inhibitor) were obtained from Selleck Chemicals (Houston, TX, USA). The study was reviewed and approved by the Institutional Review Board and the Research Ethics Committee of Shanghai General Hospital.

### RNA preparation and RT-qPCR

Total RNA was isolated using TRI Reagent® (Sigma-Aldrich; Merck KGaA) according to the manufacturer's instructions. A RevertAidTM H Minus First Strand cDNA Synthesis Kit (Thermo Fisher Scientific, Inc.) was used to reverse transcribe 1 μg of total RNA with random hexamer primers. For quantitative PCR, a LightCycler® 2.0 (Roche Diagnostics GmbH, Mannheim, Germany) and AbsoluteTM qPCR SYBR®-Green Capillary Mix (Thermo Fisher Scientific, Inc.) were used. The primers are listed in Table 1. The cycling conditions consisted of a denaturation step at 95° C for 10 min; 40 cycles of 95° C for 15 sec and annealing at 60° C for 45 sec; 95° C for 15 sec; and 60° C for 1 min. Gene expression was quantified based on the ^ΔΔ^Cq method, with β-actin as the reference housekeeping gene. The primers were produced by Shanghai Sangon Biological Engineering Technology and Services Company (Shanghai, China).

**Table 1:** Primer sequences of LDH-A and GAPDH.

**Gene name**	**Size (bp)**	**Primer sequences**
LDH-A (NM_002301.4)	191	F: 5' AGAACATGGTGATTCTAGTGTGC 3' R: 5' ACAGTCCAATAGCCCAAGAGG 3'
GAPDH (NM_001256799.1)	110	F: 5' CACCCACTCCTCCACCTTTG 3' R: 5' CCACCACCCTGTTGCTGTAG 3'

F, forward; R, reverse.

### Cell proliferation assay

Cell proliferation was evaluated using a Cell Counting Kit-8 (CCK-8) (Promega, Madison, WI, USA). SKOV3 cells and A2780 cells were seeded in a 96-well culture plates at a density of 2×10^5^ cells/well and incubated with different concentrations of the drugs (oxamate, AG14361 and olaparib) for different times. Cells treated in the absence of a test compound were the negative controls. Detection reagent was added to the cells, and the luminescence signals were determined with an EnVision™ 2100 Multilabel Reader (PerkinElmer, Santa Clara, CA, USA).

### Cell migration ability determined by scratch assay

SKOV3 cells (5×10^5^ per well) and A2780 (5×10^5^ per well) cells were inoculated in 6-well plates for 12 hours. Then, the medium was replaced with fresh medium without FBS, and the cells were incubated overnight. A scratch was made on the cell monolayer using sterile pipette tips, and phosphate-buffered saline (PBS) was used to wash away the floating cells, and then the medium was replaced with fresh medium. Oxamate (50 mM) was added, and initial photographs were taken for the first time. Then, the cells were incubated for 48 hours, and photographs were taken for a second time. The mean distance was obtained based on the cell migration distance measured by Image-Pro Plus Analysis software.

### Cell invasion ability determined by transwell assay

A total of 40 μl of Matrigel gel was dissolved at 4° C in a Transwell chamber, which had been precooled and placed in an incubator for 1 h for gelling. A cell suspension was prepared, and the cells were seeded into the upper Transwell chamber (BD Biosciences Company, USA) in a 24-well plate (2×10^4^/well for the SKOV3 and A2780 cells). The cells were incubated for 12 h. Then, the medium was replaced with fresh medium without FBS, and the cells were incubated overnight. Oxamate (50 mM) was then added. Fresh medium containing 20% FBS was added to the bottom of each well, and the cells were incubated for 48 h. At the end of the incubation period, the Transwell chambers were removed from the 24-well plates, fixed with methanol for 5 min and stained with crystal violet for 5 min. Non-invading cells on the top of the Transwell chamber were scraped off on the top of the Transwell chamber with a cotton swab. After washing with PBS, the cells were viewed under a high-power microscope (Olympus, Japan). Five visual fields were chosen, the cells that had passed through the membrane were counted, and the mean calculated.

### Western blotting

Protein was extracted from SKOV3 and A2780 cells and quantified with a Bradford assay (Bio-Rad Laboratories, Inc., Hercules, CA, USA), and 50 μg of the cleared lysates was separated on a 12% SDS-PAGE gel and electrotransferred onto PVDF membranes (EMD Millipore, Billerica, MA, USA). Actin was used as an equal loading control. The PVDF membranes were blocked in Tris-buffered saline containing 0.1% Tween-20 (TBST) with 5% nonfat dry milk for 2 h. The primary antibodies used were anti-BRCA1 (1:1000, Cell Signaling Technology, Danvers, MA, USA), anti-Cyclin B1 (1:1000, Cell Signaling Technology, Danvers, MA, USA) and anti-β-actin (1:1000, Absin Bioscience Inc., China). The membranes were then washed 3 times with PBST for 5 min each time and incubated with goat anti-rabbit IgG H&L (HRP) (1:200; cat. no. ab205718; Abcam) in PBST for 1 h. Following 3 washes with PBST, the bands were visualized using an enhanced chemiluminescence (ECL) detection system (Pierce Biotech Inc.; Thermo Fisher Scientific, Inc.) according to the manufacturer's instructions. The software that was used for densitometry was Image-Pro Plus (version 6.0; Media Cybernetics, Rockville, MD, USA).

### Tumor model

All experimental procedures and animal care were approved by Shanghai General Hospital. Eight-week-old female NMRI nude mice (Shanghai Model Organisms Center, Shanghai, China) were used to generate an A2780 xenograft model and SKOV3 xenograft model. In the animal facility, all mice were acclimatized for one week before injection of tumor cells. For establishment of xenograft tumors, 10^7^ cells were diluted in 100 μL Matrixgel™ Basement Membrane Matrix (BD Biosciences, San Jose, CA, USA) and then injected into the left flank. The tumor volumes were measured every week and survival time was recorded.

### Statistical analysis

All experiments were performed in triplicate. All statistical analyses were performed using SPSS version 22.0 software (IBM Corp., Armonk, NY, USA). T-test and one-way analysis of variance (ANOVA) were used to analyze the data between two independent groups or among three or four groups, respectively. P<0.05 was considered to indicate a statistically significant difference.
